# Overexpressing Ferredoxins in *Chlamydomonas reinhardtii* Increase Starch and Oil Yields and Enhance Electric Power Production in a Photo Microbial Fuel Cell

**DOI:** 10.3390/ijms160819308

**Published:** 2015-08-14

**Authors:** Li-Fen Huang, Ji-Yu Lin, Kui-You Pan, Chun-Kai Huang, Ying-Kai Chu

**Affiliations:** Graduate School of Biotechnology and Bioengineering, Yuan Ze University, Taoyuan 320, Taiwan; E-Mails: jylin0620@gmail.com (J.-Y.L.); pku07216@gmail.com (K.-Y.P.); hck0903@gate.sinica.edu.tw (C.-K.H.); mn32369@hotmail.com (Y.-K.C.)

**Keywords:** *Chlamydomonas reinhardtii*, ferredoxin, heat, oil, salt, starch

## Abstract

Ferredoxins (FDX) are final electron carrier proteins in the plant photosynthetic pathway, and function as major electron donors in diverse redox-driven metabolic pathways. We previously showed that overexpression of a major constitutively expressed ferredoxin gene *PETF* in *Chlamydomonas* decreased the reactive oxygen species (ROS) level and enhanced tolerance to heat stress. In addition to *PETF*, an endogenous anaerobic induced *FDX5* was overexpressed in transgenic *Chlamydomonas* lines here to address the possible functions of FDX5. All the independent FDX transgenic lines showed decreased cellular ROS levels and enhanced tolerance to heat and salt stresses. The transgenic *Chlamydomonas* lines accumulated more starch than the wild-type line and this effect increased almost three-fold in conditions of nitrogen depletion. Furthermore, the lipid content was higher in the transgenic lines than in the wild-type line, both with and without nitrogen depletion. Two FDX-overexpressing *Chlamydomonas* lines were assessed in a photo microbial fuel cell (PMFC); power density production by the transgenic lines was higher than that of the wild-type cells. These findings suggest that overexpression of either *PETF* or *FDX5* can confer tolerance against heat and salt stresses, increase starch and oil production, and raise electric power density in a PMFC.

## 1. Introduction

Photosynthetic microalgae are autotrophic cells that have rapid growth capacity; as such they are key resources in biorefineries [1]. However, the cultivation of microalgae during biomass production is sensitive to environmental factors [2,3]. To increase the biomass production of microalgae, the screening of naturally available isolates for rapid growth rates, high lipid contents, and broad environmental tolerance are all highly desirable [4]. Genetic engineering is also commonly performed to develop microalgal strains possessing these genetic traits [1,5,6].

The unicellular green alga *Chlamydomonas reinhardtii* has been used as a model organism for studying photosynthesis, starch metabolism and oil synthesis [7,8,9]. In addition, *C. reinhardtii* offers the potential to generate bioelectricity in a photo microbial fuel cell (PMFC), a device that is able to produce electrical energy using photo microorganisms under light [10]. Electrons produced by the microorganisms are transferred to the anode and flow to the cathode compartment via an external circuit [11]. The level of reduction of the photo microorganisms can be genetically engineered to improve their performance in PMFCs.

Ferredoxins (FDXs) are widely distributed iron-sulfur containing proteins and (2Fe-2S) cluster ferredoxins usually harbor the conserved amino acid motif CX_4_CX_2_CX_n_C for cluster insertion [12]. FDXs have a very negative reduction potential and therefore they function as major electron donors in diverse redox-driven metabolic pathways [13]. In plants and microalgae, FDXs deliver reducing equivalents from photosystem I (PSI) in photosynthetic organisms and provide electrons to FDX-NADPH-oxidoreductase (FNR) for NADP^+^ reduction, which is essential in the Calvin cycle for carbon assimilation [13]. FDXs can also transfer electrons to other enzymes such as ferredoxin-thioredoxin reductase (FTR) in carbon metabolism, nitrite reductases in nitrogen assimilation, and fatty acid desaturases [14,15,16,17]. These FDX-involving metabolic pathways suggest that FDXs may facilitate biomass production through the transfer of excess electrons generated from photosynthesis to enzymes involved in biomass production.

FDXs also play crucial roles in ROS (reactive oxygen species)-scavenging systems by regenerating ascorbate that protects the photosynthetic apparatus in the water-water cycle [18]. High levels of ROS cause oxidative damage of DNA, proteins, and membranes, and can lead to cell death. Chloroplasts are a prime source of ROS, which are created as byproducts of photosynthesis even in the most favorable conditions [18,19]. ROS such as hydrogen peroxide (H_2_O_2_) are further increased in cells suffering from stress due to heat or excess salt [20]. To reduce the toxicity of ROS, photosynthetic organisms have evolved several anti-oxidant systems [21]. These pathways include enzymes involved in free-radical scavenging, which in turn help relieve the oxidative stress on the cell [14,19]. The elimination of ROS toxicity is a practical approach to improve cell stress tolerance. For example, ectopic expression of cyanobacterial flavodoxin, a functional analog of FDX, in transgenic tobacco decreased the ROS level and enhanced the tolerance of the plant to heat, excessive light, cold, drought, UV radiation and iron starvation [22,23]. In addition, overexpression of *PETF,* the major form of FDX in *Chlamydomonas*, increased the proportion of reduced ascorbate under normal growth conditions and decreased the ROS level, including H_2_O_2_, enhancing the cell survival rate under heat stress [24].

In *Chlamydomonas*, there are at least six plant-type FDXs, PETF and FDX2-6 [25]. These FDX genes have different expression patterns in response to various environmental conditions. For example, PETF is constitutively expressed in a broad range of environmental conditions, whereas FDX5 is highly expressed in anaerobic conditions [25,26]. PETF is known as the major photosynthetic FDX and acts in electron transfer between photosystem I and FNR [25]. PETF and FDX5 are grouped in the same phylogenetic cluster, indicating that they are most closely related to leaf-type FDXs [16,25]. The putative protein structure of FDX5 resembles that of PETF in the position of cysteine residues for possible disulfide bond formation, and both PETF and FDX5 belong to a typical plant type 2Fe-2S protein family located in the chloroplast [16,25]. FDX5 has a reduction potential very similar to that of PETF, but is unable to reduce NADP^+^ via ferredoxin-NADP-reductase and the plastidic [FeFe]-hydrogenase HydA1, so the role of FDX5 in the anaerobic pathway is unclear [16,27].

In this study, transgenic *Chlamydomonas* cell lines overexpressing either *PETF* or *FDX5* were generated to clarify whether increasing gene expression levels of FDXs could alleviate oxidative damage under heat and salt stresses. Starch content was evaluated in these transgenic lines. Lipids accumulated more in the transgenic lines than in the wild-type cells, irrespective of nitrogen depletion. The two FDX overexpressing *Chlamydomonas* lines were applied in PMFCs; power density production by the transgenic lines was higher than that for the wild type.

## 2. Results and Discussion

### 2.1. Result

#### 2.1.1. Generation and Characterization of Transgenic Lines Overexpressing Ferredoxin Genes

**Figure 1:**
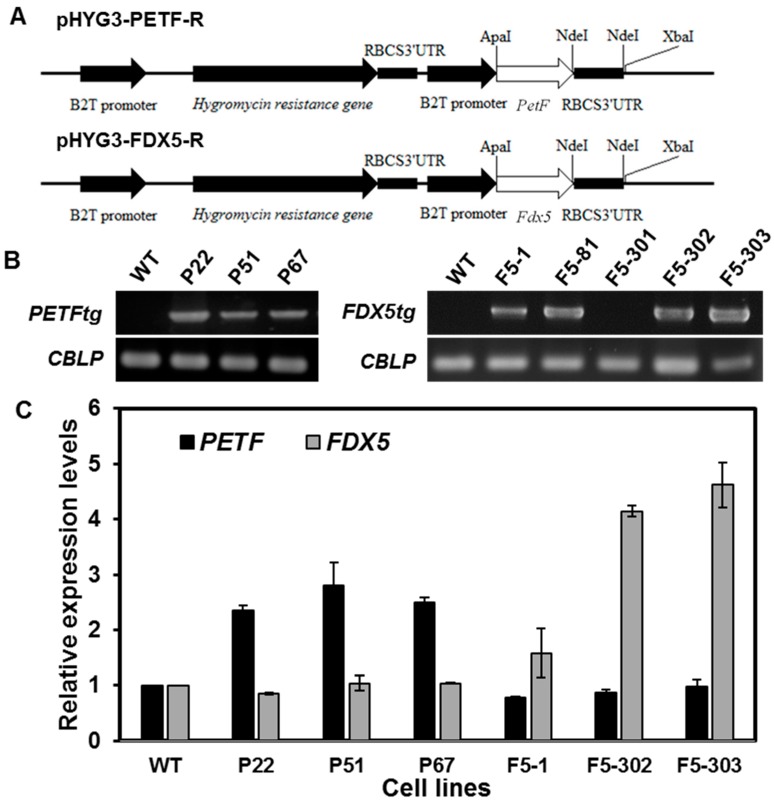
Characterization of transgenic *Chlamydomonas* overexpressing *PETF* and *FDX5*, respectively; (**A**) Schematic illustration of plasmids pHYG3-PETF-R and pHYG3-FDX5-R prepared for electroporation. The coding sequence of either *PETF* or *FDX5* was ligated between the β2-tubulin promoter (PT) and the 3ʹUTR of ribulose-1,5-bisphosphate carboxylase/oxygenase small subunit (RBCS) to generate pHYG3-PETF-R and pHYG3-Fd5-R, respectively. The transformants harboring the *aph7* gene were screened by hygromycin; (**B**) To confirm putative transformants, specific primers were used to amplify part of the promoter and the entire FDX gene to the 3ʹUTR region. Amplification of *CBLP* (G-protein beta subunit-like polypeptide) was used as an internal control for genomic DNA; (**C**) The relative quantity (RQ) of *PETF* and *FDX5* transcripts in algal lines. The total mRNA transcripts from endogenous and recombinant FDX genes, either *PETF* or *FDX5*, were quantified by qPCR with specific primers. Relative expression levels are the relative quantities of either *PETF* or *FDX5* transcripts compared to the *CBLP* transcripts respectively, and then normalized by the value of non-transformant CC125 under normal growth conditions.

The binary constructs pHyg3-PETF-R and pHyg3-FDX5-R (Figure 1A), which contain different ferredoxin cDNAs, *PETF* and *FDX5*, fused downstream of the *B2T* (β2-tubulin) promoter, were transformed into *Chlamydomonas* by electroporation, and at least three putative independent transgenic *Chlamydomonas* lines were selected for each construct. To evaluate the putative transgenic *Chlamydomonas* lines, PCR-based genomic DNA analysis was performed using specific primers to confirm the presence of transgenes. The transgene *PETFtg* was detected in the cell lines P22, P51 and P67, and *FDX5tg* was detected in lines F5-1, F5-81, F5-302 and F5-303, respectively. In contrast, no transgenes were detected in the wild-type line (Figure 1B). The *CBLP* gene, encoding a *Chlamydomonas* G-protein beta subunit-like polypeptide, was used as a loading control for the extracted genomic DNA (Figure 1B). The levels of total *PETF* and *FDX5* mRNA were determined by quantitative RT-PCR in each selected transgenic line. Figure 1C shows that *PETF* mRNA accumulated more in lines P22, P51 and P67 than in the wild type, as did *FDX5* mRNA in lines F5-1, F5-302 and F5-303. These results indicate that the selected transgenic *Chlamydomonas* lines were ectopically overexpressing either *PETF* or *FDX5*.

#### 2.1.2. Overexpression of Ferredoxins Promotes Tolerance to Heat and Salt Stresses in *Chlamydomonas*

**Figure 2:**
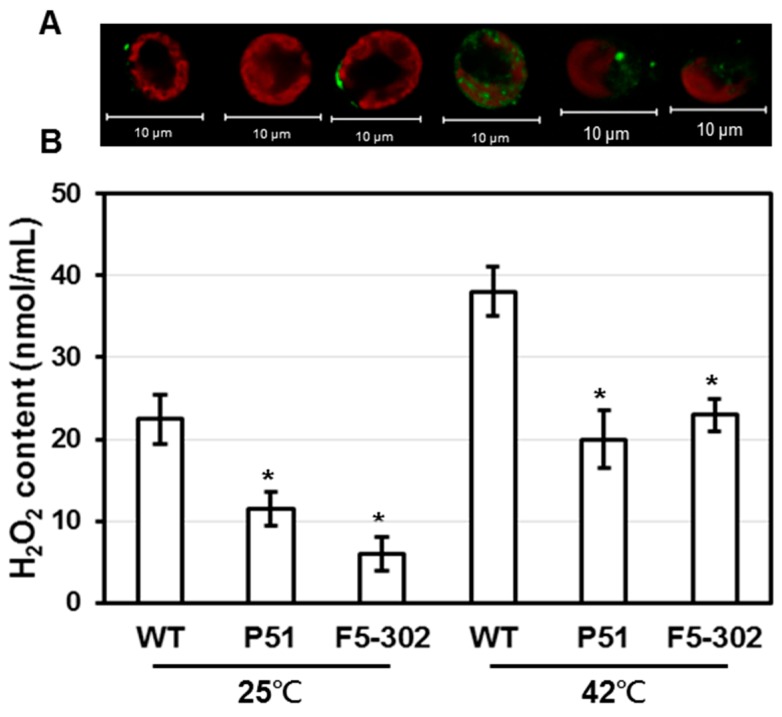
Comparison of ROS accumulation pattern and H_2_O_2_ content in wild-type and transgenic cell lines overexpressing ferredoxins under normal and high temperature growth conditions. Wild-type (WT), *PETF* transgenic line (P51), and *FDX5* transgenic line (F5-302) were incubated at 25 or 42 °C for 40 min and stained with H_2_DCFDA to monitor the accumulation of cellular ROS (**A**); The green color indicates the presence of ROS. The red color indicates autofluorescence of chlorophyll. Bars indicate 10 μm. The H_2_O_2_ content in cells was also measured (**B**). Error bars indicate SD of the mean for five replicate cell suspensions. Asterisks on the columns indicate significant differences from the WT based on Student’s *t*-test (*p* < 0.05).

In our previous study it was shown that overexpression of *PETF* in transgenic *Chlamydomonas* lines led to greater heat tolerance than in the wild type. ROS levels in these transgenic *PETF* lines also decreased in both normal and heat-stressed growth conditions [24]. To address whether overexpression of *FDX5* also lowered the ROS level in *Chlamydomonas*, the levels of H_2_O_2_, which is the major ROS, were compared in the wild-type and transgenic lines. The accumulation of H_2_O_2_ was lowered by ectopic expression of *PETF* in both normal and heat-stress conditions (Figure 2), consistent with our previous results [24]. The H_2_O_2_ levels in the *FDX5* transgenic line were lower than those in the wild-type cells in normal and heat-stressed conditions (Figure 2). A dye to label cellular ROS, H_2_DCFDA, was applied to observe *Chlamydomonas* cells under a confocal microscope. A ROS green signal was not observed under normal growth conditions for either the wild-type or transgenic lines. After heat treatment, strong ROS fluorescent signals were detected in the chloroplast of wild-type cells, whereas slight ROS signals were observed in both the *PETF* and *FDX5* overexpression lines (Figure 2). These results indicate that ectopic expression of *FDX5* cDNA decreased the ROS level in cells, similar to previous observations for ectopic expression of *PETF*.

**Figure 3:**
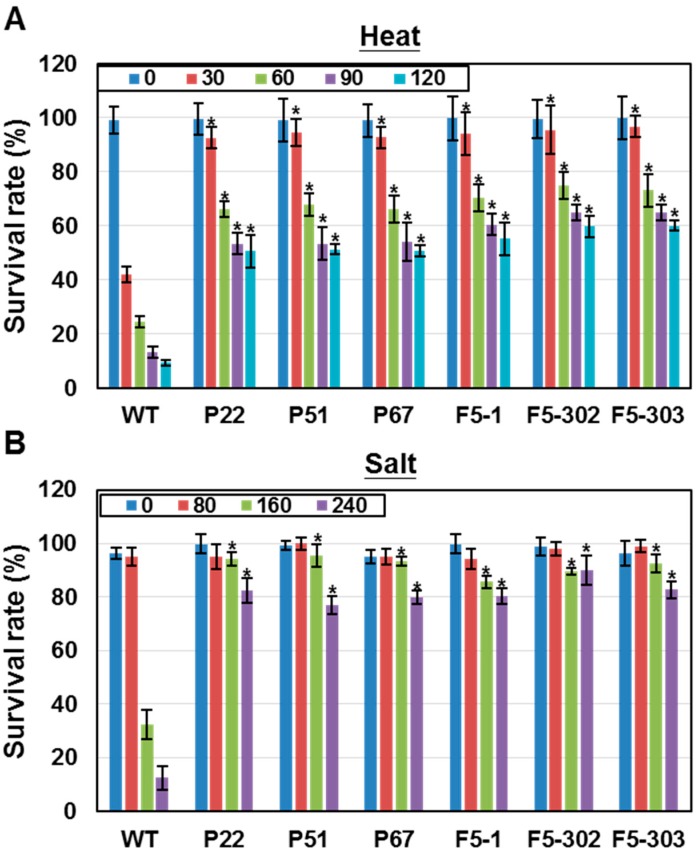
The survival rates of transgenic *Chlamydomonas* under heat and salt stresses. The wild-type (WT), *PETF* transgenic lines (P22, P51 and P67) and *FDX5* transgenic lines (F5-1, F5-302 and F5-303) of *Chlamydomonas* were treated with either heat or salt stress. (**A**) Survival rates of *Chlamydomonas* cells under heat stress. *Chlamydomonas* cell lines were cultured at 42 °C for 30, 60, 90 or 120 min, then the cells were recovered at 25 °C for 72 h and the survival rates determined; (**B**) Survival rates of *Chlamydomonas* cells under salt stress. *Chlamydomonas* cell lines were cultured in 80, 160 or 240 mM NaCl for 12 h, and the survival rates were determined. Error bars indicate SD of the mean for five biological repeats; asterisks on the columns indicate significant differences from the WT based on Duncan’s multiple range test (*p* < 0.05).

The survival rates of *PETF* and *FDX5* transgenic lines under heat stress were measured to examine whether FDX5 facilitates microalgal adaptation to heat stress, as PETF does [24]. Cells were subjected to heat treatment at 42 °C for various time periods and recovered for 3 days. The cell viability of the wild-type line dropped to 40% after heat treatment for 30 min, and progressively decreased to 10% after 120 min (Figure 3A). In contrast, the survival rates of two independent *FDX5* transgenic lines, F5-1, F5-302 and F5-303, remained at about 95% after heat treatment for 30 min (Figure 3A). Although survival rates progressively decreased in these transgenic lines as the heat treatment time increased, the *FDX5* transgenic lines remained around 60% viable after heat treatment for 120 min. Similar heat tolerance was also observed in three independent *PETF* transgenic lines, P22, P51 and P67 (Figure 3A).

Salt (NaCl) stress have been reported to increase the levels of ROS and inhibit the cell growth in *Chlamydomonas* [28]. To examine whether overexpression of *PETF* or *FDX5* conferred salt tolerance in transgenic *Chlamydomonas* lines, the *PETF* and *FDX5* transgenic lines were treatment with 80, 160 and 240 mM salt concentrations. The survival rate of the wild-type line dropped to 35% and 15% after incubation in 160 and 240 mM NaCl for 12 h, respectively, whereas the survival rates of *PETF* and *FDX5* transgenic lines remained at 80%–90% (Figure 3B). Taken together, these results indicate that the overexpression of *PETF* or *FDX5* genes enhances the tolerance of *Chlamydomonas* to heat and salt stresses.

#### 2.1.3. Overexpression of Ferredoxins Promotes Tolerance to Heat and Salt Stresses in *Chlamydomonas*

**Figure 4:**
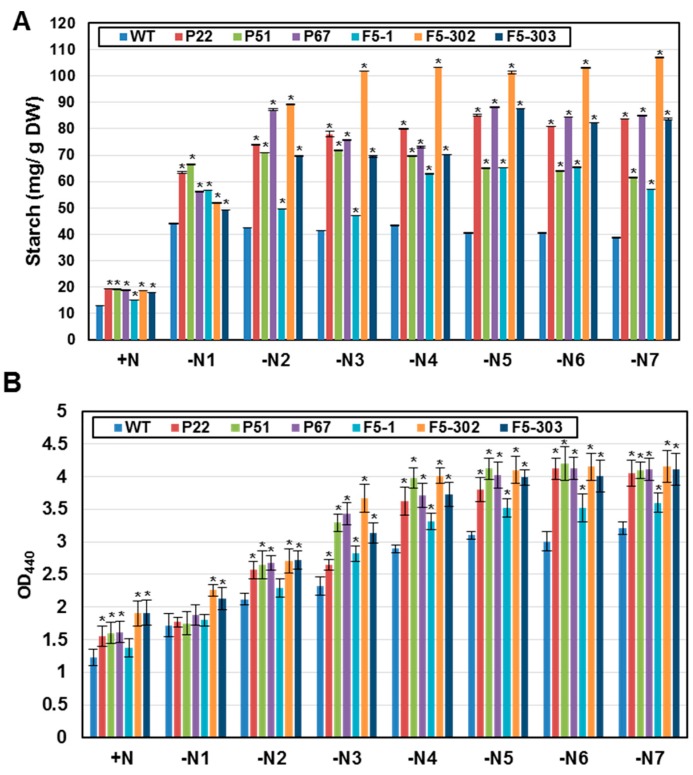
Accumulated starch and lipid in two ferredoxin gene transgenic lines. Wild type (WT), *PETF* transgenic lines (P22, P51 and P67), and *FDX5* transgenic lines (F5-1, F5-302 and F5-303) were grown in nitrogen-containing medium (+N) or nitrogen-free medium for 1 to 7 days (−N1 to −N7). Cells were collected and then (**A**) starch and (**B**) lipid contents were determined. Error bars indicate the SDs of five independent biological replicates and asterisks on the columns indicate significant differences from the WT based on Duncan’s multiple range test (*p* < 0.05).

FDX is proposed to play a role in starch metabolism [29], and starch is known to accumulate during nitrogen depletion in *Chlamydomonas* [30]. To test whether ectopic expression of *FDXs* increased starch accumulation under nitrogen starvation, we measured the starch contents of *PETF* and *FDX5* overexpressing transgenic lines after nitrogen starvation for various periods. The starch content increased by 60% relative to wild type in transgenic lines overexpressing either *PETF* or *FDX5* in normal growth conditions (Table 1; Figure 4A). Nitrogen starvation-induced starch accumulation was observed in the wild-type line and in the transgenic lines (Table 1; Figure 4A). Compared to the wild type, *PETF* or *FDX5* transgenic lines contained 1.7–2.7 times more starch after nitrogen starvation for three to seven days (Table 1; Figure 4A). These results indicated that overexpression of *FDXs* could enhance starch accumulation in *Chlamydomonas*.

**Table 1:** Starch contents (mg/g dry weight) of WT, *PETF*- and *FDX5*-overexpression lines.

Line	+N	−N1	−N2	−N3	−N4	−N5	−N6	−N7
WT	13.05 ± 0.06	44.13 ± 0.05	42.50 ± 0.05	41.33 ± 0.03	43.33 ± 0.09	40.53 ± 0.04	40.53 ± 0.06	38.77 ± 0.13
*PETF* overexpression lines								
P22	19.40 ± 0.15	63.40 ± 0.30	73.91 ± 0.12	77.96 ± 1.06	80.00 ± 0.24	85.08 ± 0.34	80.82 ± 0.03	83.69 ± 0.11
P51	19.13 ± 0.03	66.46 ± 0.07	70.82 ± 0.08	71.74 ± 0.12	69.69 ± 0.16	65.14 ± 0.30	63.84 ± 0.10	61.43 ± 0.24
P67	18.86 ± 0.08	56.08 ± 0.11	87.19 ± 0.38	75.71 ± 0.12	73.07 ± 0.24	88.26 ± 0.21	84.34 ± 0.02	84.90 ± 0.06
*FDX5* overexpression lines								
F5-1	15.04 ± 0.05	56.85 ± 0.01	49.59 ± 0.07	47.14 ± 0.09	62.89 ± 0.15	65.25 ± 0.10	65.25 ± 0.16	57.11 ± 0.08
F5-302	18.60 ± 0.02	52.97 ± 0.18	89.13 ± 0.18	101.79 ± 0.11	103.30 ± 0.10	101.31 ± 0.62	103.21 ± 0.18	106.88 ± 0.10
F5-303	18.11 ± 0.01	49.22 ± 0.10	69.77 ± 0.19	69.52 ± 0.36	70.31 ± 0.07	87.54 ± 0.01	82.25 ± 0.12	83.69 ± 0.21

+N indicates cells cultured in nitrogen-containing medium; −N1 to −N7 indicates cells cultured in nitrogen-free medium for one to seven days. Error bars indicate the SDs of five independent biological replicates.

Oil accumulation was also compared among wild type, *PETF* and *FDX5* transgenic lines. Equal cell numbers of wild-type, two *PETF* transgenic lines, and two *FDX5* transgenic lines, were incubated in nitrogen depletion medium for various periods, and relative lipid contents were compared. Quantitative assays showed that the lipid content was increased 13%–56% in P22, P51, P67, F5-302 and F5-303 cells compared with wild-type cells in normal growth conditions (Table 2; Figure 4B). After nitrogen starvation, lipids progressively increased in both the wild-type and transgenic lines; the increase ranged from 0.5 to 2.5 folds from day 1 to day 4 of nitrogen starvation (Table 2; Figure 4B), but the lipid levels remained approximately constant from day 4 to day 7 (Table 2; Figure 4B). These results indicate that both overexpression of *PETF* and *FDX5* can promote oil accumulation in *Chlamydomonas* in either nitrogen-rich or -starved conditions.

**Table 2:** Relative lipid contents (OD_440_) of WT, *PETF* and *FDX5* overexpression lines.

Line	+N	−N1	−N2	−N3	−N4	−N5	−N6	−N7
WT	1.22 ± 0.12	1.72 ± 0.17	2.12 ± 0.08	2.31 ± 0.14	2.89 ± 0.06	3.10 ± 0.06	3.00 ± 0.15	3.21 ± 0.10
*PETF* overexpression lines								
P22	1.56 ± 0.16	1.77 ± 0.07	2.57 ± 0.13	2.65 ± 0.08	3.63 ± 0.22	3.80 ± 0.19	4.12 ± 0.16	4.05 ± 0.20
P51	1.60 ± 0.16	1.75 ± 0.17	2.65 ± 0.21	3.29 ± 0.13	3.98 ± 0.16	4.12 ± 0.16	4.20 ± 0.25	4.10 ± 0.12
P67	1.61 ± 0.16	1.88 ± 0.15	2.68 ± 0.11	3.43 ± 0.17	3.71 ± 0.19	4.0 ± 0.20	4.13 ± 0.17	4.11 ± 0.16
*FDX5* overexpression lines								
F5-1	1.38 ± 0.14	1.80 ± 0.09	2.30 ± 0.14	2.82 ± 0.11	3.31 ± 0.13	3.52 ± 0.14	3.52 ± 0.21	3.60 ± 0.14
F5-302	1.90 ± 0.19	2.26 ± 0.09	2.71 ± 0.19	3.67 ± 0.22	4.01 ± 0.12	4.10 ± 0.21	4.15 ± 0.21	4.16 ± 0.25
F5-303	1.91 ± 0.19	2.12 ± 0.17	2.72 ± 0.14	3.13 ± 0.16	3.72 ± 0.19	3.99 ± 0.12	4.01 ± 0.24	4.11 ± 0.25

+N, indicate cells were cultured in nitrogen-containing medium; −N1 to −N7, indicate cells were cultured nitrogen-free medium for 1 to 7 days. Error bars indicate the SDs of five independent biological replicates.

#### 2.1.4. Application of Transgenic *Chlamydomonas* Ferredoxin Lines in a Photo Microbial Fuel Cell

**Figure 5:**
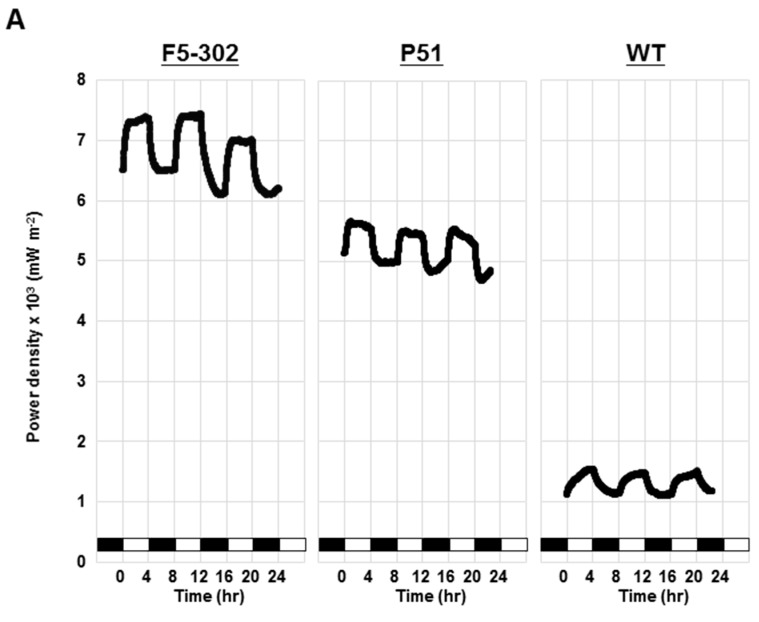

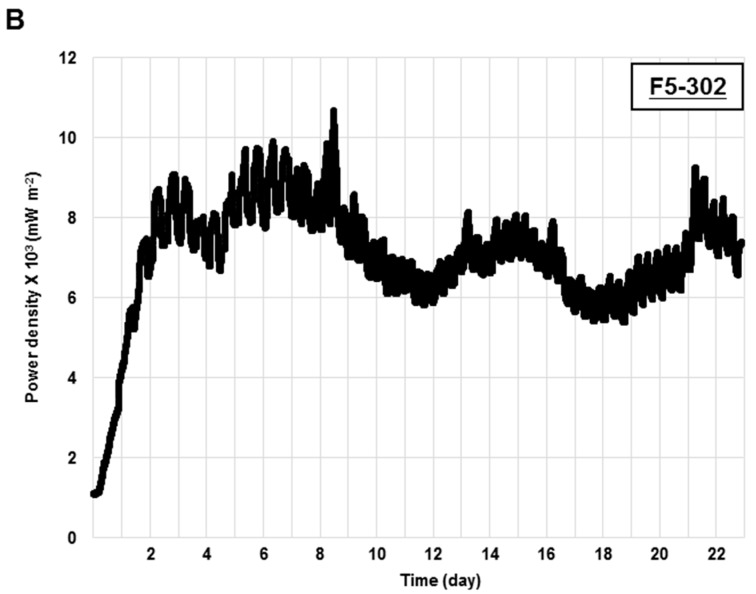
Electricity generated from *Chlamydomonas* in a photo microbial fuel cell (PMFC). (**A**) *FDX5* transgenic line F5-302, *PETF* transgenic line P51, and Wild type (WT) were individually assessed in a PMFC containing 80 mM NaCl under a 4 h/4 h light/dark cycle. White/black bars indicate the light/dark cycle; (**B**) The power density generated by F5-302 was monitored for 23 days.

FDXs are electron transfer proteins responsible for delivering reducing equivalents from photosystem I [13]. Electricity generation in photo microbial fuel cells (PMFC) was compared for the wild type, the *PETF* overexpression line P51 and the *FDX5* overexpression line F5-302. Equal cell number of *Chlamydomonas* at the late-logarithmic growth phase was loaded in the anaerobic anode chamber and operated under 4 h light/4 h dark photoperiods. Electric current and voltage were detected every minute and the power density was calculated. The power density rose during light periods and declined during dark periods in all three *Chlamydomonas* lines (Figure 5A). The maximum power density generated by F5-302 was 0.0084 mW·m^−2^, and P51 was 0.0055 mW·m^−2^, both higher than the wild-type line (0.0015 mW·m^−2^) (Figure 5A). Meanwhile, light-dependent power density production was more effective in F5-302 than P51 and wild type (Figure 5A). Furthermore, long-term power density production from the F5-302 line in PMFC was examined. Mid-logarithmic *Chlamydomonas* cells were loaded in the anaerobic anode chamber and operated under 6 h light/6 h dark photoperiods for seven days to increase cell number, and then the photoperiod was switched to 4 h light/4 h dark. The power density was 0.001 mW·m^−2^ on day zero, and then increased progressively to 0.009 mW·m^−2^ by day three (Figure 5B). After day three, the power density maintained at a range of 0.0058–0.01 mW·m^−2^ for more than three weeks (Figure 5B), and light-dependent power density phenomena was detected in long-term PMFC with the F5-302 *Chlamydomonas* transgenic cell line (Figure 5B).

### 2.2. Discussion

Microalgae are considered a primary resource for third-generation biofuels, because they can produce biomass rapidly with simple cultivation requirements. Some microalgal strains can even be directly cultivated with flue gas and wastewater [31,32]. In order to use microalgae in biofuel production, scientists are screening strains highly tolerant to stressful environmental conditions. Under various stresses, excess ROS accumulates in cells, causing oxidative damage and generally leading to cell death. Thus, reduction of ROS level is a major biotechnology strategy to protect photosynthetic organisms from various abiotic stresses [33,34,35]. In adverse environmental conditions such as drought, salt, high temperature and excessive light, the main source of ROS production is in chloroplasts via the photosynthetic electron-transport chain (PETC) due to uneven electron flow. Therefore, avoidance of excess electrons overproduced by the PETC in stressed cells may reduce ROS production and prevent cellular damage. FDXs are electron distributers that transfer electrons originating from photosynthetic water oxidation to specific FDX-dependent enzymes in the chloroplast. In addition, FDXs can also transfer electrons to generate ascorbate, which is employed by ascorbate peroxidase (APX) to scavenge H_2_O_2_ [19,21,36,37]. Thus, overexpression of FDXs is a theoretical method to reduce ROS and improve abiotic stress tolerance. Recent evidences indicated that PETF and FDX5 directly interact with the cyanobacterial flavoprotein Flv3 homolog protein [38]. Flv3, a NAD(P)H: oxygen oxidoreducase in *Sybechocystis* sp., has been proposed to play a role in detoxification of oxidative compounds [39]. Previously, we demonstrated that overexpression of *PETF*, a major form of ferredoxin, decreases the ROS level and contributes to tolerance to heat stress in *Chlamydomonas* [24]. Protein binding properties between PETF and FDX5 have been demonstrated by a yeast-two hybrid system and pull-down experiments [38], implying a functional interaction between PETF and FDX5. In the present study, we provide evidence that ROS levels in the *Chlamydomonas* transgenic lines overexpressing either *PETF* or *FDX5* were significantly reduced, resulting in improved tolerance to heat and salt stresses. Our investigation supports the hypothesis described above that the overexpression of FDXs decreases ROS levels and contributes to tolerance to abiotic stresses in *Chlamydomonas*.

Microalgae have chloroplasts that contain light-harvesting antenna complexes (LHCs) to capture light energy, and chloroplasts generate ROS during energy transfer steps of photosynthesis [40]. Down-regulation of LHC related proteins reduces photosynthetic efficiency. Accordingly, the reduction in expression of LHC in *Chlamydomonas* by RNAi technology results in a higher resistance to photodamage, and increases light penetration properties compared to that in non-transgenic cells [41]. FDXs accept electrons generated from photosystems and then transfer them to specific FDX-dependent enzymes. PETF is shown to interact physically with the light-harvesting protein LHCA5, involved in the formation of the LHCI super complex [38]. In the present study, we propose that *PETF* overexpression lines may transport excess electrons from photosystems more effectively than the nontransgenic line, reducing ROS levels and also preventing photoinhibition, thereby increasing the starch content. Given that FDX5 interacts with PETF, the results described in the discussion above in FDX5 overexpression lines may be mediated by PETF.

In this study, transgenic lines of *Chlamydomonas* overexpressing either the *PETF* or the *FDX5* gene all showed higher starch accumulation than the wild type. In *Chlamydomonas*, PETF, the most abundant ferredoxin, has been shown to interact directly with FNR1, which can reduce NADP^+^ to the NADPH that is later used in the Calvin Cycle to assimilate CO_2_ [38,42]. Moreover, PETF and FDX5 can also interact directly with the starch branching enzymes (SBE2/3) [38] involved in starch synthesis. In addition, through interaction with FDX thioredoxin reductase (FTR), ferredoxin proteins donate electrons to thioredoxins (Trx) that activate ADP glucose pyrophosphorylase (AGPase), a Trx target enzyme, by formation of a disulfide bond between the two small subunits of the AGPase heterotetrameric complex, a key enzyme in starch synthesis [43,44]. Both PETF and FDX5 have been shown to interact with chloroplastic FTR [38].

Several enzymes, such as pyruvate dehydrogenase and fatty acid synthase, require ATP and NAD(P)H to run fatty acid biosynthesis [12]. The transgenic lines in this study had significantly higher lipid contents than the wild type. This could be linked with FDXs promoting NADPH production and CO_2_ assimilation, in turn, to increase lipid accumulation. Moreover, *in vitro* activity analyses indicated that FDXs provide electrons to fatty acid desaturases [45,46]. We will further investigate the mechanism of lipid enhancement by the *PETF*- and *FDX5*-overexpressing lines in future work.

Microbial fuel cells (MFCs) are a microbial electricity generation system that produces electric power from catalytic reactions of organic substrates and biomass. The design of better materials for MFCs, the optimization of conditions for growth and activity of microbial cells, and the screening of more electrochemically active microbes, are all areas of work that have been undertaken in a bid to enhance the generation of electric power by MFCs [11,47,48,49,50,51,52]. The photomicrobial fuel cells (PMFCs) by using photo-microorganisms can generate electro energy under photosynthesis without supplement of carbon source. Both *PETF* and *FDX5* overexpression transgenic lines were applied in a PMFC and the electric power density was higher than for wild-type cells, indicating that these FDX transgenic lines have high electrochemical activities. It is possible that overexpression of FDXs can enhance electron transfer to the anode of the PMFC. Another possibility is that the FDX transgenic lines accumulate more biomass, such as starch, which can be converted into cytosolic NADPH by the pentose phosphate pathway. In turn, electrons transfer to the extracellular space by NADPH oxidases encoded by *RBO1* (respiratory burst oxidase) and *RBO2* [10] (Anderson *et al.*, 2015). Interestingly, the *FDX5* transgenic line exhibited higher electric power density than the wild-type line and *PETF* transgenic lines, implying that *FDX5* is a major electron carrier in *Chlamydomonas* in anaerobic conditions when *FDX5* is induced and FDX5 is stabled [16]. Future studies are required to determine the action of FDX5 in the PMFC.

## 3. Experimental Section

### 3.1. Cultivation of Chlamydomonas reinhardtii

The wild-type strain of *Chlamydomonas reinhardtii* (CC125) and transgenic *PETF* or *FDX5* overexpression lines were grown in Tris-acetate-phosphate (TAP) liquid medium [53] for a 12 h photoperiod (with a light intensity of 125 µmol photons·m^−2^·s^−1^) at 25 °C. The cell density was adjusted to 3 × 10^6^ mL^−1^, and 1 mL of cell suspension was added into 99 mL of TAP with shaking at 150 rpm for liquid culture.

### 3.2. Construction of Plasmids

To construct a plasmid for constitutive expression of the *FDX5* gene, the *FDX5* cDNA fragment was amplified by reverse transcription polymerase chain reaction (RT-PCR) with primers cFDX5-F: 5ʹ-ACGGGCCCATGCTGTGCGCGCGCTCCCAG-3ʹ (*Apa*I site underlined) and cFDX5-R: 5ʹ-GCCATATGTTACTGGTGCTTGCCGTACTCG-3ʹ (*Nde*I site underlined). The PCR fragment was digested with *Apa*I and *Nde*I, and ligated into pHYG3-PETF [24] to generate pHYG3-FDX5, which contains the β*2*-*tubulin* promoter driving expression of the *FDX5* gene. As a terminator, the 3ʹ-UTR of the *rbcS2* gene (ribulose bisphosphate carboxylase small subunit) was amplified by PCR with rbcS3-F: 5ʹ-AACATATGCGCTCCGTGTAAATGGAGGC-3ʹ (*Nde*I site underlined) and rbcS3-R: 5ʹ-AACATATGTCTAGACGCTTCAAATACGCCCAGC-3ʹ (*Nde*I site underlined), digested with *Nde*I and ligated with pHYG3-FDX5 to produce pHYG3-FDX5-R. The same construction strategy was applied to produce plasmid pHYG3-PETF-R for expression of PETF.

### 3.3. Transformation of Chlamydomonas reinhardtii

*C. reinhardtii* strain CC125 cells were electroporated according to a method described previously [24]. Both pHYG3-PETF-R and pHYG3-FDX5-R were linearized by *Sca*I and then transferred into a 4 mm gap electroporation cuvette, respectively. For electroporation, the Gene Pulser Xcell™ system (Biorad, Hercules, CA, USA) was used, with parameters 2 kV·cm^−1^, 25 µF, and 500 Ω. Cells were screened on TAP plates containing 20 μg·mL^−1^ hygromycin. All putative *PETF* and *FDX5* transgenic cell lines were confirmed by genomic PCR with specific primers to amplify the recombinant DNA fragment including the *β-tubulin* (B2T) promoter, FDX coding sequence and the *rbcS2* terminator region (RBCS 3ʹUTR). These primers were B2TP-F (5ʹ-CTAGATCACTACCACTTCTACACAG-3ʹ) and P721 (5ʹ-GCGTATCACGAGGCCCTTTC-3ʹ).

### 3.4. Heat and Salt Treatment

For heat stress treatment, log phase of cells were centrifuged and then 3 × 10^6^ mL^−1^ cells were cultured in TAP medium. One mL of cell suspensions were subjected to treatment at 42 °C under light (125 µmol photons·m^−2^·s^−1^) for various periods, and then recovered for 72 h under continuous light at 25 °C. Cell survival rate was determined by trypan blue staining analysis. The cell suspension was mixed with an equal volume of 0.4% (*w*/*v*) trypan blue solution. The survival rate was determined by hemocytometer and calculated as (unstained living cell number)/(total cell number) × 100%.

For salt stress treatment, log phase of cells were centrifuged and then 3 × 10^6^ mL^−1^ cells were cultured in TAP medium containing different NaCl concentrations (0, 120, 160 and 240 mM) under light condition (125 µmol photons·m^−2^·s^−1^) for 12 h. Cell survival rate was determined as described as heat stress treatment.

### 3.5. Quantitative RT-PCR

Total RNA was extracted from log phase of *Chlamydomonas* cells (total 3 × 10^7^ cells) using a plant total RNA kit (Viogene, Taipei, Taiwan). Then, cDNA fragments were synthesized from 1 μg of total RNA using the Transcriptor First Strand cDNA Synthesis Kit (Roche, Penzberg, Germany). To measure the total *PETF* and *FDX5* transcripts in PETF-transgenic, FDX5-transgenic and wild-type lines, quantitative PCR was performed using a PikoReal™ 96 Real-Time PCR System (Thermo Scientific, Waltham, MA, USA). Primers PETF-F (5ʹ-GCTATGCGCTCCACCTTC-3ʹ) and PETF-R (5ʹ-CGTCCAGGATGTAGGTGTCA-3ʹ) were designed to amplify *PETF* transcripts. Primers FDX5-F2 (5ʹ-CGGGCAAGACGAAGACTATGG-3ʹ) and FDX5-R2 (5ʹ-GGGTAGGCGAGCACATGAG-3ʹ) were designed to amplify *FDX5* transcripts. *CBLP* (G-protein beta subunit-like polypeptide) transcripts were analyzed as an internal control using primers Re-Cblp-F and Re-Cblp-R as previously described [24].

### 3.6. Detection of H_2_O_2_ and Reactive Oxygen Species (ROS)

For H_2_O_2_ measurement, an Amplex red hydrogen peroxide/peroxidase assay kit (Invitrogen, Carlsbad, CA, USA) was used. Log phase *Chlamydomonas* cells (total 3 × 10^6^) were collected by centrifugation and then frozen in liquid nitrogen, ground with a steel ball, and dissolved in 100 μL of 1 × reaction buffer from the assay kit. The mixture was centrifuged at 13,000× *g* for 5 min, and then the supernatant was used to measure cellular H_2_O_2_ concentration after horseradish peroxidase treatment at 25 °C for 30 min. The H_2_O_2_ concentrations were determined using a standard curve plotted from 0.2 to 1.0 μM H_2_O_2_ standards. Data were collected with five repeats.

To detect ROS, cells were incubated with 10 μM 2ʹ,7ʹ-dichlorodihydrofluorescein diacetate (H_2_DCFDA) and then observed using a Zeiss LSM 510 META confocal laser-scanning microscope (Carl Zeiss Microscopy GmbH, Jena, Germany). Fluorescent signals emitted from H_2_DCFDA and autofluorescence of chlorophyll were excited at 488 nm, and emitted signals were obtained at 500–530 and 650–710 nm, respectively.

### 3.7. Starch Quantitation

Total 3 × 10^7^ *Chlamydomonas* cells in log phase were cultured in 100 mL TAP and nitrogen starved-TAP medium for various periods. Cells were harvested by centrifugation and then freeze-dried. Sample dry weight was measured and then cells were resuspended in 80% (*v*/*v*) ethanol at least three times to remove chlorophyll, judged by the loss of green color. Samples were dried again at 70 °C and resuspended in 50 mM HClO_4_ to measure starch contents, as described previously [54].

### 3.8. Lipid Quantitation

To rapidly evaluate lipid levels, a colorimetric quantitation from algal cultures was applied as described [55], with slight modification. A total of 3 × 10^7^ *Chlamydomonas* cells in log phase were cultured in 10 mL TAP and nitrogen starved-TAP medium for various periods. Cells were pelleted by centrifugation and frozen at −20 °C. Whole cells were thawed in 200 μL of saponification reagent (25% methanol in 1 N NaOH), lysed with a stainless-steel ball and then heated evenly in a boiling water bath for 30 min. For colorimetric detection of cellular lipids, 200 μL of neutralization reagent (1 N HCl, 100 mM Tris pH 8.0) and 200 μL of copper reagent ((1 M triethanolamine):(1 *N*-acetic acid):(6.45% (*w*/*v*) Cu (NO_3_)_2_·3H_2_O) = 9:1:10) were added to each sample, and then mixed with 250 μL of chloroform. After centrifugation, the organic phase was obtained and then subjected to relative lipid content measurement as described [55].

### 3.9. Photo Microbial Fuel Cell System (PMFC)

A dual-chambered microbial fuel cell was fabricated using transparent poly-acrylic plastic plates. The two chambers were separated by a Nafion 211 membrane (Du Pont, Wilmington, DE, USA). The volume of each chamber was 70 mL. Two electrodes were graphite plates (each of 45.09 cm^2^) that were installed horizontally at opposite side of PMFC and a platinum wire was applied to connect the chambers to conduct electricity. Before the PMFC was set, the membrane and electrodes were pre-treated as described by Raman and Lan (2012); the electrodes were pre-treated with 1.0 M HCl for 1 h followed by 1.0 M NaOH for 1 h; the Nafion 211 membrane was pre-treated by sequentially boiling it in H_2_O_2_ (30% *v*/*v*) and deionized water, followed by soaking in 0.5M H_2_SO_4_ and then deionized water, each for 1 h. The anode chamber contained 3 × 10^8^ harvested algal cells in TAP medium with 80 mM NaCl, and was continuously stirred at 250 rpm using a magnetic stirrer under anaerobic condition. The cathode solution contained 0.1 M K_3_Fe(CN)_6_, as an electron acceptor, with 0.5 M K_2_HPO_4_ and 80 mM NaCl at pH 7. The PMFC was operated under 4 h of light (~30 µmol photons·m^−2^·s^−1^) and 4 h of darkness. The voltage and current across the external resistor (1 kΩ) were measured at 1 min intervals using a dual-channel data collection multi-meter (PROVA, Taipei, Taiwan). Power density (PD, mW·m^−2^) was calculated as PD = IV/area, where P = power, I = current (A), V = voltage (V).
